# The efficacy of intensity modulated radiation therapy in treating thyroid-associated ophthalmopathy and predictive factors for treatment response

**DOI:** 10.1038/s41598-017-17893-y

**Published:** 2017-12-13

**Authors:** Yong-Jiang Li, Yong Luo, Xiao-Qi Xie, Wei-Min He, Cheng Yi, Ping Li, Feng Wang

**Affiliations:** 1 Department of Oncology, West China Hospital, Sichuan University, Chengdu, PR China; 2 Department of Critical Care Medicine, West China Hospital, Sichuan University, Chengdu, PR China; 3 Department of Ophthalmology, West China Hospital, Sichuan University, Chengdu, PR China

## Abstract

The study evaluated clinical efficacy of intensity modulated radiation therapy (IMRT) in treating patients with thyroid-associated ophthalmopathy (TAO) and defined predictive factors that associated with treatment response. A total of 178 TAO patients were treated with retro-orbital IMRT with radiation dose of 20 Gy in 10 fractions. The immediate and long-term treatment response and complications were evaluated. Besides, logistic-regression analysis was conducted to identify possible predictive factors. TAO symptom score significantly fell from the initiation to 6-month post-treatment (P < 0.001). 134 patients (73.2%) had mild to significant response to IMRT, and 172 patients (96.6%) achieved stabilization of TAO without future progression. Current smoker (OR 2.88, 95% CI 1.32–6.29; P = 0.008) and symptom duration longer than 18 months (OR 3.33, 95% CI 1.24–8.93; P = 0.017) were identified as independent predictive factors for non-response of TAO to retro-orbital IMRT. Immediate complications were slight and self-limited, and long-term complications mainly included chronic xerophthalmias in12 patients (6.74%) and cataract formation in 4 patients (2.25%). The study suggested that IMRT was a viable option for treating TAO patients, with a satisfactory symptom control ability and acceptable post-treatment complications.

## Introduction

Thyroid-associated ophthalmopathy (TAO), an inflammatory fibrosing pathology of retro-orbital contents, is the most common extrathyroidal manifestation of autoimmune hyperthyroidism (Graves’ disease). It is an autoimmune disease caused by production of auto-antibodies that activate the thyroid stimulating hormone receptors (TSHR), which is primarily located in thyroid tissues but also expressed in adipose and connective tissues within the retro-orbital area. These auto-antibodies induced inflammatory and fibrotic responses of the retro-orbital fat and extraocular muscles (EOMs), characterized by T cell infiltration and fibroblastic glycosaminoglucan accumulation^1–3^. The subsequent edema of the EOMs and fats increased volume within the bony orbital cone, and led to typical symptoms of TAO including orbital pain, proptosis, tearing, EOM dysfunction, diplopia, sight loss, exposure keratitis, corneal ulceration and even blindness.

The treatment TAO aimed at preserving vision, reducing diplopia, relieving orbital pain and improving cosmetic appearance. For patients whose TAO symptoms are not relieved after normalization of thyroid function, glucocorticoids have been traditionally considered as the first-line treatment method. About 65% of these patients could benefit from the corticoids, but relapse of TAO symptoms is common when the corticoids are reduced or withdrawn^4,5^. Besides, the usage of corticoids is also restricted by its multiple side-effects including hyperglycemia, hypertension and immune system compromise.

Radiation therapy (RT) is an established modality for the treatment of TAO, and is particularly useful in treating patients who are insensitive to or cannot tolerate corticoids^6^. In fact, there are previous reports concluding that RT should be used as the first-line therapy for TAO, although controversy still existed in the field^7,8^. The mechanism of RT in treating TAO is mainly through its non-specific anti-inflammatory effects, suppressing radiosensitive lymphocytes and inhibiting fibroblast proliferation and glycosaminoglucan secretion^9^. Despite RT is slower than corticoids to reveal its curative effects, it could provide a more prolonged protection period^10^.

Traditionally, lateral opposing fields (LOF) technique has been utilized by radiation oncologists in treating TAO for its simple set-up and prompt delivery procedures. However, one of the most obvious drawbacks of the technique is that, to minimize the dose to lenses, beams are either blocked to anterior portion of the globes or titled 5 degrees posteriorly, which would lead to inadequate dose to the EOM insertions and anterior portion of the retro-orbital fats that are commonly involved in TAO process. Besides, dose distribution within the target is hard to reach homogeneous by the LOF technique. Afterwards, new techniques including three-dimensional conformal radiotherapy (3DCRT) and intensity modulated radiation therapy (IMRT) emerged, and have been regarded as the current standard radiation technique for head and neck tumors. Nevertheless, the clinical outcomes of using these techniques in treating TAO patients have not been reported.

IMRT, as an evolutionary form of 3DCRT, is capable to deliver a dose distribution around a more irregular and complex target volume. Besides, it could achieve steeper dose gradients between the target and normal structures, thus reducing the dose to surrounding tissues without compromising the planning target coverage^11–13^. As a result, IMRT may be more applicable in the retro-orbital radiation because of the quite irregular target volume of the retro-orbital structures. Indeed, Lee *et al*. identified the dosimetric superiority of IMRT in the retro-orbital radiation in his dosimetric study involving 10 TAO patients undergoing IMRT treatment^14^. IMRT was found to have a significantly superior conformity index and homogeneity index than 3DCRT and LOF, and could provide better dose sparing to globes, lenses and optic nerves. Nevertheless, as it is a dosimetric study, no treatment outcomes were reported, and the number of patients undergoing IMRT was limited. Moreover, no other studies has been done concerning the clinical outcomes of the retro-orbital IMRT in treating TAO patients.

Therefore, the primary goal of the current study is to evaluate the therapeutic efficacy of the retro-orbital IMRT in treating TAO, as well as the accompanying acute and long-term complications; the secondary goal is to identify possible predictive factors that associate with the treatment response to the retro-orbital IMRT.

## Methods

Our research is in accordance with the Helsinki Declaration and approved by the medical ethics committee of West China Hospital, Sichuan University. Written informed consent has been obtained from all individual participants included in the study.

The study is a retrospective study based on prospective collection of treatment data. The data of disease severity, therapeutic response and complications were prospectively collected in clinic when we treated TAO patients with retro-orbital IMRT technique between July 2010 and December 2014. Patients were included in the study with the following inclusion criteria: (1) Patients were diagnosed with TAO. The diagnosis of TAO was confirmed at the Department of Ophthalmology, West China Hospital after a complete neuro-ophthalmologic examination. The full workup included history and physical examination, thyroid disease history, thyroid laboratory tests, and CT and/or MRI imaging used to rule out other diseases including infection and tumor. (2) The TAO was treated with IMRT technique at our department. (3) Medical records and laboratory reports of the patients were complete. (4) The patients had regular follow-up visits and the corresponding clinic data were available. Patients were excluded in the following conditions: (1) lost to follow-up or died because of other diseases; (2) had received radiotherapy for TAO or decompressive surgery before enrollment; (3) treated with other radiotherapy techniques including LOF and 3DCRT; (4) only patients with hyperthyroidism were included to achieve a more homogenous background, those with hypothyroidism were excluded; and also, (5) patients with single-eye irradiation were excluded.

Patients’ TAO symptoms were classified into 5 general categories according to the NOSPECS system: soft tissue signs, proptosis, EOM dysfunction, corneal involvement, and sight loss^15^. However, due to several limitations of the NPSPECS system^16^, a more comprehensive TAO symptom evaluation system was utilized in the current study, which included additional symptom categories of diplopia, orbital pain and tearing in evaluation^17^. Each category, except for the tearing, was assigned a score of 0, 1 or 2 representing no symptoms, mild to moderate symptoms, and severe symptoms, respectively. Tearing was allocated a score of 0 (no tearing) or 1 (tearing). Total TAO symptom score was the cumulative score of all the listed categories and ranged from 0 to 15.

Treatment response of the TAO symptom was based on the score difference between the baseline score and 6-month post-treatment score, which was calculated as [(baseline score – 6-month post-treatment score)/baseline score], and categorized as follows: >66% - significant response, 33% to 66% - moderate response, 10% to 33% - mild response, 0% to 10% - no response and <0% - progression. Treatment response in the individual symptom categories was defined as complete response (CR) if the symptom was completely resolved, or partial response (PR) if the symptom was improved but not completely resolved.

**Figure 1:**
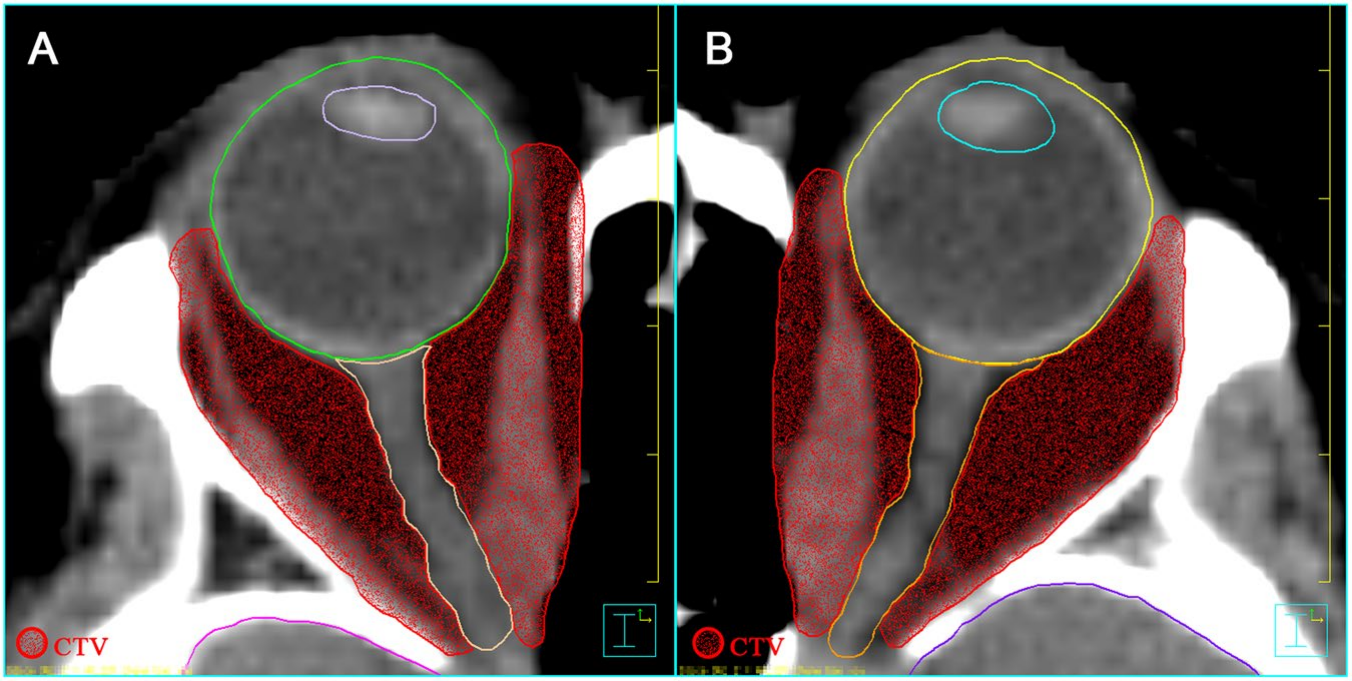
Target volume delineation (**A**,**B**) of IMRT in treating TAO.

**Figure 2:**
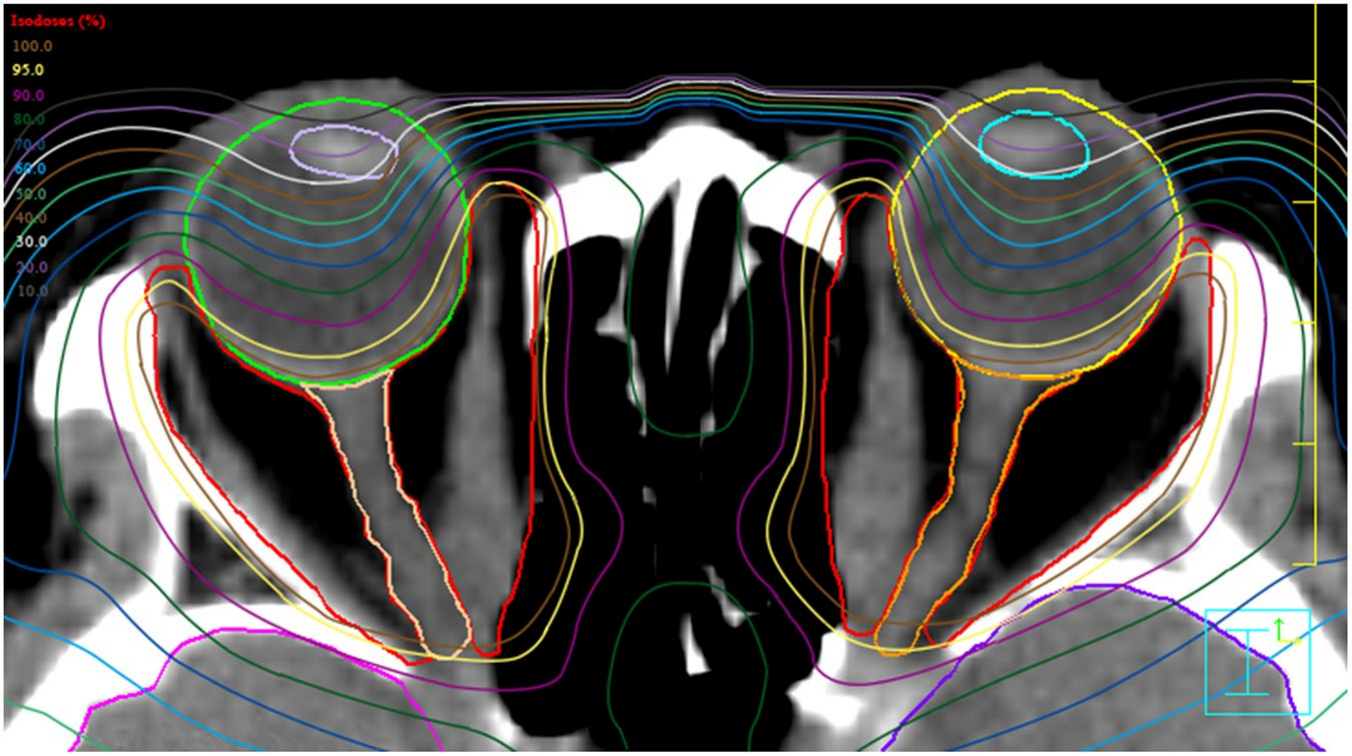
Planned dose distribution of IMRT in treating TAO.

All patients received retro-orbital radiation with linear accelerator based step-and-shoot IMRT technique. Patient was immobilized with a custom-made thermoplastic mask, and then they underwent CT scan with slice sickness of 2.5 mm for image acquisition and target contouring. The origins to insertions of the EOMs and the retro-orbital fatty spaces within the main bulk were encompassed as the clinical target volume (CTV) (Fig. 1A,B). Lacrimal glands, lenses, globes and optic nerves were outlined as organs-at-risk (OAR). A 2 mm concentric margin around the CTV area was generated as planning target volume (PTV). All the patients received a total dose of 20 Gy in 10 fractions within two to three weeks delivered by reversely planned 7-filed IMRT. The IMRT plans were verified to ensure that 90% isodose line cover the PTV, and then the beams were generated by 6MV linear accelerator. Figure 2 illustrated a typical planned dose distribution. After radiotherapy, the patients were followed-up by radiation oncologist and ophthalmologist bimonthly for the first half-year, then every 3 months for the first year, and biannually thereafter unless a specific clinical event emerged.

The statistical analyses were performed using SPSS Statistics version 22.0. Descriptive statistics were utilized to characterize the patient population, treatment outcomes and complications. The symptom scores at different treatment periods were compared by Kruskal-Wallis test and Mann-Whitney U test, and illustrated in Box plots. Pearson’s Chi-squared test and Fisher’s exact test were conducted to identify the variables predictive of non-response to IMRT, and a multivariate logistic-regression analysis was performed to determine the independent risk factors. All the statistical analyses were considered significant at two-tailed P < 0.05.

### Data availability statement

The datasets generated during and/or analysed during the current study are available from the corresponding author on reasonable request. All data generated or analysed during this study are included in this published article.

### Ethical approval

All procedures performed in studies involving human participants were in accordance with the ethical standards of the institutional and/or national research committee and with the 1964 Helsinki declaration and its later amendments or comparable ethical standards.

## Results

### Patients’ characteristics

The characteristics of these patients were presented in Table 1. A total of 178 patients were enrolled in the study, including 105 female and 73 male patients. The median age was 54 years (range 22–82 years), and the median follow-up period was 53 months (range 30–83 months). A total of 149 patients (83.7%) had previous treatment for hyperthyroidism, including medication treatment in 134 patients (75.3%), radioactive iodine in 70 patients (39.3%) and thyroidectomy in 22 patients (12.4%). Sixty-four patients (35.9%) had previous steroid use for TAO, of whom 46 patients (71.9%) had mild to moderate response and 18 patients (28.1%) had no response. The duration of TAO symptoms before RT was less than 6 months in 68 patients (38.2%), 6 to 18 months in 75 patients (42.1%) and longer than 18 months in 35 patients (19.7%). During the radiotherapy, 42 patients (23.6%) had concurrent steroid use and the rest patients (n = 136, 76.4%) received radiotherapy only. For the smoking status, 97 patients were non-smokers (54.5%) while 19 patients (10.7%) were former smokers and 62 patients (34.8%) were current smokers. The symptom scores were 1 to 5 in 47 patients (26.4%), 6 to 10 in 102 patients (57.3%) and 11 to 15 in 29 patients (16.3%).

**Table 1:** Basic characteristics of patients.

Variables	No.	%
Gender		
Female	105	59.0%
Male	73	41.0%
Median age	54 years (range 22–82)	
Previous thyroid treatment		
Medication	134	75.3%
Thyroidectomy	22	12.4%
RAI	70	39.3%
None	29	16.3%
Smoking status		
Non-smoker	97	54.5%
Former smoker	19	10.7%
Current smoker	62	34.8%
Duration of TAO prior to RT		
≤6 months	68	38.2%
6–18 months	75	42.1%
>18 months	35	19.7%
Previous steroid use		
Yes	64	35.9%
No	114	64.1%
Response to previous steroids		
None	18	28.1%
Mild to moderate	46	71.9%
Significant	0	0%
Concurrent steroid use during RT		
Yes	42	23.6%
No	136	76.4%
Symptom severity scores at enrollment		
1 to 5	47	26.4%
6 to 10	102	57.3%
11 to 15	29	16.3%

*Abbreviations:* RAI, radioactive iodine; TAO, thyroid-associated ophthalmopathy; RT, radiation therapy.

### Immediate response

**Figure 3:**
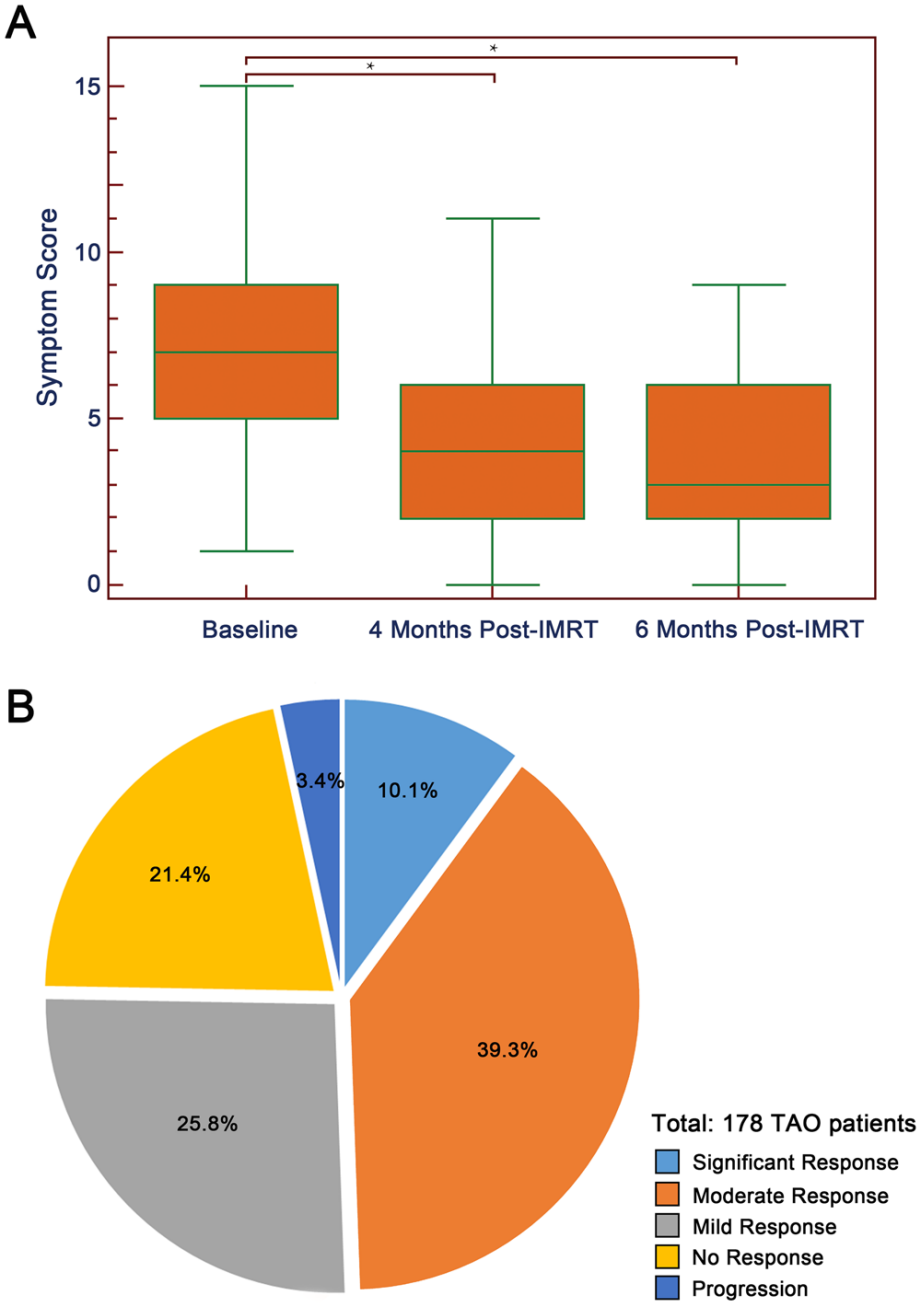
Treatment response to IMRT in the initial 6 months. (**A**) The changes in TAO symptom score; (**B**) The percentage of patients in different response degrees.

The immediate response of the whole symptom severity to IMRT has been shown in Fig. 3. The median symptom scores at the enrollment, 4 months and 6 months post-IMRT were 7 (range 1–15), 4 (range 0–11) and 3 (range 0–9), respectively. The 4-month score (P < 0.001) and 6-month score (P < 0.001) were significantly lower than the initial score, while the difference between 4-month score and 6-month score did not reach statistically significant (P = 0.397) (Fig. 3A). For the patients, majority of them experienced improvement of TAO symptoms, and more specifically, the overall treatment response was mild in 46 patients (25.8%), moderate in 70 patients (39.3%) and significant in 18 patients (10.1%). For the rest patients, 38 of them (21.3%) showed no response to retro-orbital IMRT and 6 patients (3.3%) experienced progression of the TAO symptoms (Fig. 3B).

The response to IMRT in the categories of individual symptoms at 6-month post-treatment was shown in Table 2. Orbital pain had the best treatment reaction, with CR rate of 71.6% and improvement rate (CR & PR) of 82.4%. Besides, EOS dysfunction and tearing also had favorable treatment response, with CR rates of 31.7% and 36.8%, and improvement rates of 53.8% and 78.2%, respectively. Besides, soft tissue involvement had a relatively high improvement rate of 63.8%. In contrast, proptosis and sight loss was the most refractory symptoms, with CR rates of 10.7% and 20.5%, and improve rates of 45.0% and 46.2%, respectively.

**Table 2:** Response to IMRT by category of symptoms.

Category	No. (%)	No. CR	Percentage	No. CR & PR	Percentage
Soft tissue involvement	127 (71.3)	22	17.3	81	63.8
Proptosis	131 (73.6)	14	10.7	59	45.0
EOM dysfunction	104 (58.4)	33	31.7	56	53.8
Corneal involvement	10 (5.6)	3	30.0	5	50.0
Sight loss	39 (24.9)	8	20.5	18	46.2
Orbital pain	74 (41.6)	53	71.6	61	82.4
Tearing	87 (48.9)	32	36.8	68	78.2
Diplopia	72 (40.5)	17	23.6	37	51.4

*Abbreviations:* PR, partial response; CR, complete response; EOM, extra-ocular muscle.

In the aspect of acute complications, slight hair loss around sideburns and increased milphosis or madarosis were the most common complications after retro-orbital IMRT, which occurred in 26 patients (14.6%) and 32 patients (17.9%), respectively. The symptoms commonly appeared from 1-month to 4-month post-IMRT, and could be in remission spontaneously by 6 months after the treatment. Unilateral or bilateral eye redness also occurred in 12 patients (6.74%), within the period from start of radiotherapy to 4 months post-treatment. Similarly, the eye redness could be spontaneously relieved or eliminated within a week. Particularly, a total of 21 patients (11.8%) informed of increased orbital pain accompanying with self-consciously progressed proptosis after the treatment, but their symptoms achieved PR or CR at the 6 months’ follow-up visit. This may be caused by temporary edema of the retro-orbital tissues after RT.

### Long-term response

The symptom score of these patients at the last follow-up was not significantly different from the 6-month post-treatment score, with the same median score of 3 points. For the 134 patients who had immediate treatment reactions of mild, moderate and significant response, only 7 patients (5.2%) had obvious exacerbation or recurrence of TAO symptoms, and the disease of the rest 127 patients (94.8%) was maintained or continued to improve. It needs to mention that, 5 of the 7 patients with recurrent TAO symptoms were current smokers and refused to quit smoking. For the 44 patients with immediate treatment reactions of no response or progression, no obvious improvement of the disease severity happened during the subsequent follow-ups, and surgery intervention was recommended by ophthalmologists to those meeting the operation indication.

As for complications, the intermittent eye redness, slight sideburns hair loss, and increased milphosis or madarosis were not presented in the cohort at the last follow-up visit. A total of 12 patients (6.74%) developed chronic xerophthalmias, and were well-controlled with administration of artificial tears. Cataracts occurred in 4 patients (2.25%), and were treated successfully with cataract removal and lens replacement. No radiation retinopathy or secondary malignancy has been detected in the cohort.

### Predictive factors for non-response to retro-orbital IMRT

Table 3 showed the results of univariate analysis for various factors predictive of non-response to retro-orbital IMRT. Factors that associated with non-response to IMRT included current smoker (OR 2.98, 95% CI: 1.42–6.28; P = 0.004) duration of TAO symptoms longer than 18 months (OR 3.45, 95% CI: 1.36–8.79; P = 0.009), and male sex (OR 2.08, 95% CI: 1.04–4.15; P = 0.037). A tendency of concurrent steroid use towards treatment response could be found, but it did not reach statistically significant (OR 0.43, 95% CI: 0.17–1.12; P = 0.079). The multivariate analysis demonstrated that the current smoker (OR 2.88, 95% CI: 1.32–6.29; P = 0.008) and duration of TAO symptoms longer than 18 months (OR 3.33, 95% CI: 1.24–8.93; P = 0.017) remained independent predictive factors for non-response of TAO symptoms to retro-orbital IMRT (Table 4).

**Table 3:** Univariate analysis on predictive factors for non-response to retro-orbital IMRT.

Characteristics	Odds ratio	95% CI	p-value
Age (>55 vs. ≤55)	1.57	0.79–3.13	0.195
Sex (Male vs. Female)	2.08	1.04–4.15	0.037
Duration of TAO prior to RT			
≤6 months	reference		
6–18 months	1.76	0.77–4.03	0.182
>18 months	3.45	1.36–8.79	0.009
Symptom severity scores at enrollment			
1 to 5	reference		
6 to 10	0.73	0.33–1.57	0.416
11 to 15	0.61	0.21–1.84	0.384
Previous steroids use (Yes vs. No)	1.50	0.75–3.01	0.251
Response to previous steroids (PR vs. None)	0.71	0.22–2.30	0.563
Concurrent steroid use during RT (Yes vs. No)	0.43	0.17–1.12	0.079
Smoking status			
Non-smoker	reference		
Former smoker	1.81	0.57–5.73	0.314
Current smoker	2.98	1.42–6.28	0.004

*Abbreviations:* CR, complete response; TAO, thyroid-associated ophthalmopathy; RT, radiation therapy.

**Table 4:** Multivariate analysis on predictive factors for non-response to retro-orbital IMRT.

Characteristics	Odds ratio	95% CI	p-value
Sex (Male vs. Female)	1.98	0.95–4.13	0.066
Concurrent steroid use during RT (Yes vs. No)	0.45	0.16–1.27	0.102
Duration of TAO prior to RT			
≤6 months	reference		
6–18 months	1.69	0.71–4.03	0.231
>18 months	3.33	1.24–8.93	0.017
Smoking status			
Non-smoker	reference		
Former smoker	1.75	0.53–5.78	0.355
Current smoker	2.88	1.32–6.29	0.008

*Abbreviations:* CR, complete response; TAO, thyroid-associated ophthalmopathy; RT, radiation therapy.

## Discussion

To our knowledge, the current study based on 178 patients with TAO firstly evaluated the overall efficacy and treatment outcomes of retro-orbital IMRT in treating TAO symptoms, and identified possible risk factors predictive of non-response to the treatment method. Our findings demonstrated that IMRT was a feasible option for treating TAO patients, with a satisfactory disease control ability and acceptable post-RT complications. In addition, current smoking and duration of TAO longer than 18 months were identified as independent risk factors for poor response of TAO to IMRT.

Overall, the retro-orbital IMRT exhibited satisfactory therapeutic effects in our cohort. The total symptom score significantly fell from the initiation of the treatment to 4-month post-RT. Although the 6-month score was not statistically different from the 4-month score, a decreasing trend could still be found. In the aspect of patients, 73.2% of them had mild to significant response to IMRT, and 96.6% of them achieved stabilization of TAO without future progression. Compared with previous studies utilizing LOF technique, the proportions in the current cohort are at a relatively high level^6,17–19^. All our patients received a total dose of 20 Gy in 10 fractions to both retro-orbital contents, which is the most adopted radiation protocols in previous studies. However, it should be noted that the 20 Gy/10-fraction protocol was established as a balance of efficacy and toxicity based on LOF technique. In other words, 20 Gy in 10 fractions was identified by previous reports as the most efficacious and minimally toxic treatment dose in treating TAO patients when using the LOF technique. As IMRT could achieve a better dose differentiation between tumor target and normal tissues, the protocol may not be the most optimized protocol for IMRT. Considering that it has not been established that whether 20 Gy was adequate for the retro-orbital contents to reach plateau of relief, IMRT may provide a chance to further raise the dose of the target volume to achieve a higher symptom control ability without causing more complications, and the optimal RT protocol is needed to be clarified in future studies.

In the category of individual symptoms, the response to radiotherapy exhibited heterogeneity. It has been established in previous studies with NPSPECS system that RT primarily affected soft tissue involvement and EOM dysfunction^7,17^, and our results were in accordance with the previous studies that the two symptom categories had relatively high improvement rates (Table 2). As we additionally evaluated diplopia, orbital pain and tearing, we found that the orbital pain and tearing were the most responsive symptoms to IMRT, which had the highest CR rates and improvement rates. In contrast, the proptosis and sight loss were the most refractory symptoms. The variations in response by symptom category indicated that clinicians should take the constellation of presenting symptoms into consideration while selecting patients for radiotherapy. The patients with predominantly proptosis and sight loss should be aware that they may not be able to have satisfactory benefits from radiotherapy. Besides, for patients with rapid visual deterioration, as the acute optic neuropathy requires rapid treatment to avoid irreversible damage, retro-orbital radiotherapy may not be suitable and surgical decompression should be timely considered.

We also conducted statistical analysis to identify possible predictive factors for non-response to retro-orbital IMRT. The duration of symptoms longer than 18 months was found to be significant predictive factor both in the univariate analysis and multivariate analysis. In previous studies^6,17^, although not reaching statistically significant, a trend of longer disease duration towards less probability of response to radiotherapy could be observed. The insignificance may be due to the number of patients and the difference in cut-off values of symptom duration. The underline mechanism may be correlated with the inflammation process of retro-orbital contents. Once inflammatory infiltration is replaced by fibrosis, radiotherapy is less effective in relieving the symptoms. As a result, radiotherapy should be given at early start of the TAO symptoms, especially during the active phase, to achieve a better therapeutic response.

Smoking also remained an independent risk factor for non-response in the cohort. Prior studies have shown that tobacco consumption is correlated not only with an increased risk, but also the severity of TAO in patients with Grave’s disease^20,21^. Besides, the response of TAO to steroids was significantly better in non-smokers than smokers^22^. The mechanism of the tight correlation between smoking and TAO is still largely unclear, but the elevated thiocyanate and smoking-induced hypoxia, which involve in immune reactions and release of cytokines in the retro-orbital space, have been suggested^23,24^. Our results identified current smoking as a considerable risk factor for unfavorable treatment response to retro-orbital radiotherapy. Besides, 5 out of 7 patients with recurrent TAO symptoms during the follow-ups were current smokers, indicating that smoking may also influence the efficacy maintaining periods of retro-orbital RT and the prognosis of patients. Thus, TAO patients who are current smokers should be intensively informed about the unfavorable risks and suggested to quit smoking.

Concerning the role of steroid use in retro-orbital RT, controversy still existed. Some studies reported that patients who respond to prior steroid were more possible to respond to retro-orbital RT^25^. In addition, concurrent steroid use during RT was shown to provide more benefit over RT alone in some investigations^26,27^. However, other studies showed similar curative effects between RT group and RT plus corticoids group, and moreover, a higher rate of moderate to severe complications was observed in the combined therapy group^6–8,17^. In the current study, we were also unable to show significant correlations of the prior steroid use, the response to the prior steroid use and the concurrent steroid use with the therapeutic response to retro-orbital IMRT. Thus, our results did not find an additional beneficial effect of steroids administration on retro-orbital IMRT. Nevertheless, for the patients with acute onset, high severity or rapid deterioration of TAO symptoms, steroids are possibly beneficial in rapidly symptom control and should be timely administrated.

Overall, retro-orbital IMRT was well tolerated in our patients, as no patient need treatment break due to acute toxicity. The intermittent eye redness, mild sideburns hair loss and increased milphosis or modarosis were the most common acute complications, all of which were generally slight and temporary. The relative low doses applied in treating TAO and high accuracy of IMRT may explain the mild acute toxicity. Particularly, it needs to mention that 21 patients (11.8%) in the cohort experienced self-conscious aggravation of TAO symptoms after the start of retro-orbital IMRT, but the aggravated symptoms disappeared afterwards. The pseudo-progression mostly occurred in patients with higher levels of initial symptom severity scores, and it is possibly caused by the temporary edema of the retro-orbital tissues after radiotherapy.

In the aspect of long-term complications, our study suggested satisfactory safety of IMRT in treating TAO. Only 4 patients (2.25%) in the cohort developed cataracts. Considering that the advancing age was also correlated with increased risk for cataract, the possibility of cataract directly caused by IMRT was actually further less. The low dose in treating TAO is one of the reasons. According to previous data from orbital lymphoma patients, RT-induced cataract formed after a single fraction of 200 cGy^28^. For patients who received a lens dose of 1500 cGy fractioned during a treatment course, the probability of cataract formation was 12% at 5 years^18^. Secondly, that IMRT could provide a well preservation to adjacent normal structures may also contribute to the low rate of cataract formation. With appropriate field volume designed to protect the lens, the lens dose was estimated to be under 700 cGy, fractioned in 10 fractions during 2 to 3 weeks. Thus, the risk of cataract development after IMRT for TAO is supposed to be small.

Because cataract is easily to be dealt with eye operation, some other possible complications are clinically more severe. Radiation retinopathy is a sight-limiting complication induced by ophthalmic RT, which is characterized by vascular closure, vascular incompetence and resultant loss of vision^29^. The risk of radiation retinopathy is tightly associated with total dose, dose rate, use of radiation sensitizers (e.g. chemotherapeutic agents) and the presence of systematic disease (e.g. diabetes)^29,30^. The radiation dose which can cause up to 5% radiation retinopathy within 5 years was estimated to be 45 Gy^28^. The radiation dose in our patients is much less than 45 Gy and chemotherapy is not needed, thus it is reasonable that no patients in our cohort suffered from radiation retinopathy. Secondary malignancy is another major concern. The excess lifetime risk of RT-induced cancer after treating TAO was calculated to be 0.7% in previous reports^31^, and the estimation was based on LOF technique. IMRT could significantly reduce the dose sparing to bone marrow and brain, however, the radiation dose in the painting area was larger than the conventional irradiation as IMRT used multiple fields with different intensity modulation for each beam. The soft tissues or muscles within or near the target area were susceptible to the development of radiation-induced secondary malignancy. Besides, some dosimetric studies reported increased risk of secondary malignancy in soft tissues which were exposed to low-dose radiation brought by IMRT. As a result, although no secondary malignancy was detected in our cohort, the risk of secondary malignancy development when utilizing IMRT technique in treating TAO patients should be paid attention and re-evaluated in future studies.

## Conclusion

In conclusion, the study demonstrated that retro-orbital IMRT was a viable option for treating TAO patients, with a satisfactory symptom control ability and acceptable complications. In addition, current smoking and duration of TAO longer than 18 months were identified as independent risk factors for poor response of TAO to IMRT.

### Informed consent

Informed consent was obtained from all individual participants included in the study.
